# Effects of environmental exposure to iron powder on healthy and elastase-exposed mice

**DOI:** 10.1038/s41598-024-59573-8

**Published:** 2024-04-21

**Authors:** Thiago Tafarel Galli, Elaine Cristina de Campos, Leandro do Nascimento Camargo, Silvia Fukuzaki, Tabata Marayama dos Santos, Sara Sumie Sobral Hamaguchi, Suellen Karoline Moreira Bezerra, Fabio José Alencar Silva, Bianca Goulart Rezende, Fernanda Tenório Quirino dos Santos Lopes, Clarice Rosa Olivo, Beatriz Mangueira Saraiva-Romanholo, Carla Máximo Prado, Edna Aparecida Leick, Christine Laure Marie Bourotte, Isabela Judith Martins Benseñor, Paulo Andrade Lotufo, Renato Fraga Righetti, Iolanda Fátima Lopes Calvo Tibério

**Affiliations:** 1 Faculdade de Medicina (FMUSP), São Paulo, Brazil; 2 Hospital Sírio-Libanês, São Paulo, Brazil; 3 Hospital Alemão Oswaldo Cruz, São Paulo, Brazil; 4 Institute of Geosciences - University of São Paulo (USP), São Paulo, Brazil; 5 Federal University of São Paulo (UNIFESP), São Paulo, Brazil; 6 University of São Paulo, Av. Dr. Arnaldo, 455 – Cerqueira César, São Paulo, SP 01246-903 – Laboratory LIM20 Brazil

**Keywords:** Biomarkers, Risk factors

## Abstract

Prolonged exposure to iron powder and other mineral dusts can threaten the health of individuals, especially those with COPD. The goal of this study was to determine how environmental exposure to metal dust from two different mining centers in Brazil affects lung mechanics, inflammation, remodeling and oxidative stress responses in healthy and elastase-exposed mice. This study divided 72 male C57Bl/6 mice into two groups, the summer group and the winter group. These groups were further divided into six groups: control, nonexposed (SAL); nonexposed, given elastase (ELA); exposed to metal powder at a mining company (SAL-L1 and ELA-L1); and exposed to a location three miles away from the mining company (SAL-L2 and ELA-L2) for four weeks. On the 29th day of the protocol, the researchers assessed lung mechanics, bronchoalveolar lavage fluid (BALF), inflammation, remodeling, oxidative stress, macrophage iron and alveolar wall alterations (mean linear intercept-Lm). The Lm was increased in the ELA, ELA-L1 and ELA-L2 groups compared to the SAL group (p < 0.05). There was an increase in the total number of cells and macrophages in the ELA-L1 and ELA-L2 groups compared to the other groups (p < 0.05). Compared to the ELA and SAL groups, the exposed groups (ELA-L1, ELA-L2, SAL-L1, and SAL-L2) exhibited increased expression of IL-1β, IL-6, IL-10, IL-17, TNF-α, neutrophil elastase, TIMP-1, MMP-9, MMP-12, TGF-β, collagen fibers, MUC5AC, iNOS, Gp91phox, NFkB and iron positive macrophages (p < 0.05). Although we did not find differences in lung mechanics across all groups, there were low to moderate correlations between inflammation remodeling, oxidative stress and NFkB with elastance, resistance of lung tissue and iron positive macrophages (p < 0.05). Environmental exposure to iron, confirmed by evaluation of iron in alveolar macrophages and in air, exacerbated inflammation, initiated remodeling, and induced oxidative stress responses in exposed mice with and without emphysema. Activation of the iNOS, Gp91phox and NFkB pathways play a role in these changes.

## Introduction

Environmental pollution is a major cause of disease, death, and disability in many countries worldwide. The World Health Organization (WHO) estimates that pollution causes approximately seven million deaths per year in large cities and industrial centers around the world^1^. The increase in pollutant emissions has the potential to harm the health of exposed populations by causing, for example, irritations, allergies, respiratory disease and cancer^2^.

The activity of the steel industry is a global concern, the iron ore pelletization process and its transportation, as well as coal, steel and other metals, result in increased emissions of particulate matter (PM) and ozone^3,4^.

Atmospheric particulate matter (PM) is a mixture of tiny particles and liquid droplets ranging in size from coarse to fine to ultrafine. Coarse PM particles range in diameter from 10 to 2.5 μm (PM_10_). Fine particles have a diameter of less than 2.5 μm (PM_2.5_), while ultrafine particles have diameters of less than 0.1 μm. The composition of PM varies depending on where it is collected from and may contain remnants of fuel combustion such as polycyclic aromatic hydrocarbons (PAHs), sulfates, nitrates, microbes, and chemical elements such as iron, zinc, silicon, sodium, and aluminum^5–7^.

The harm caused by PM to the lungs increases as the particle size decreases^6^. Small-sized particles remain suspended in the atmosphere for longer periods, increasing the likelihood of inhalation, and induce greater pulmonary resistance and inflammatory responses, increasing the risk of chronic lung inflammation and reducing lung function^8–11^.

Chronic obstructive pulmonary disease (COPD) is a heterogeneous lung condition characterized by chronic respiratory symptoms (dyspnea, cough, expectoration, and exacerbations) due airway dysfunction (bronchitis and bronchiolitis) and/or alveolar dysfunction (emphysema). Inflammatory responses to inhaled toxins cause these abnormalities, which results in persistent, often progressive, airflow obstruction^12^. It is now known that exposure to cigarette smoke, hazardous particles, or gases triggers an inflammatory chain reaction that produces many potent cytokines and chemokines, resulting in chronic inflammation and tissue destruction in COPD patients. However, the impact of exposure to iron ore powder on COPD patients has yet to be determined^13^.

Industrialization and urbanization has drastically reduced the air quality in metropolitan regions^2^. Atmospheric particles are made up of road dust resuspension, emissions from industrial activities, and marine aerosols. Depending on the area, particulate matter contains high levels of aluminum and silicon, which are typically found in soil. However, PM may also contain higher levels of iron, chlorine, sodium, magnesium, calcium and other trace metals^1^.

Environmental conditions have a substantial impact on the dispersion or accumulation of pollutants^14^. Temperature, relative humidity, wind speed and direction, and other variables are all directly related to air pollution and may vary with the seasons (summer and winter). Pollutant levels tend to rise when the relative humidity and wind speed are low. Furthermore, precipitation and an increase in wind speed contribute to the dispersion and dilution of pollutants, resulting in a reduction in their concentration^13^.

Accordingly, the purpose of this study was to assess the effects of environmental exposure to iron dust and fine particles on healthy mice and elastase-exposed mice in different locations (vivarium and place of exposure) in two seasons with environmental differences (summer and winter).

## Materials and methods

The present study was submitted and approved by the Review Board for Human and Animal Studies of the School of Medicine of the University of São Paulo (n° 919–17). Male C57Bl/6 mice (25–30 g, 7 weeks old, specific pathogen-free [SPF]-grade) were obtained from the Animal Facility of the School of Medicine of the University of São Paulo. Animals were^15^ maintained in cages with controlled temperature (22 ± 2 °C), humidity (70–75%), and dark/light cycle (12 h; lights on at 06:00 am) conditions and allowed food and water ad libitum. All animal care and experimental procedures followed the Guide for the Care and Use of Laboratory Animals, and all animal experiments were conducted in accordance with ARRIVE guidelines^15,16^.

### Animals

The study involved 72 mice divided into two exposure periods (36 animals exposed in summer and 36 in winter). In each period group, the animals were divided into six groups of six animals each: (1) SAL (6 animals): animals that received intratracheal instillation of saline and were kept in a vivarium in São Paulo; (2) ELA (6 animals): animals that received intratracheal instillation of elastase and were kept in a vivarium in São Paulo; (3) SAL-L1 (6 animals): animals that received intratracheal instillation of saline and were exposed to Place 1 in Vitória; (4) ELA-L1 (6 animals): animals that received intratracheal instillation of elastase and were exposed to Place 1 in Vitória; (5) SAL-L2 (6 animals): animals that received intratracheal instillation of saline and were exposed to Place 2 in Vitória; and (6) ELA-L2 (6 animals): animals that received intratracheal instillation of elastase and were exposed to Place 2 in Vitória.

The animals from the SAL and ELA groups were kept in an animal care facility at the Medical School of the University of São Paulo. The temperature and humidity in the facility were controlled, and the cages where the animals were housed had prefilters and high-efficiency particulate air filters that filtered particles greater than or equal to 0.3 µm and removed microscopic contaminants from the air.

The exposure groups were sent to the Vitória sites in the summer and winter. They were continuously exposed to the environmental factors present in these specific locations for a period of four weeks. The animals were kept in cages with controlled temperature, no sun exposure, a standard light/dark cycle. They were given food and water ad libitum and were exposed to the outside air through natural ventilation, without air filtration or decontamination. During this period, they were carefully monitored to assess the lung response and the effects of the environment on lung function and structure. Upon completion of the exposure period, the animals were carefully transported back to the city of São Paulo, in the state of São Paulo (SP), Brazil.

### Elastase-induced emphysema model

C57Bl/6 mice were anesthetized with ketamine (40 mg/kg) and xylazine (5 mg/kg) via muscular injection. To administer the porcine pancreatic elastase (PPE) (0.667 IU mixed with 50 μL of sterile saline; elastase type I/E-1250, Sigma‒Aldrich, St. Louis, MO, USA) to the lungs, the trachea was exposed, and PPE was injected directly through it before chest compression (ELA, ELA-L1, and ELA-L2 groups). Using the same procedure, the control groups received 50 μL of sterile saline (SAL, SAL-L1, and SAL-L2 groups)^17,18^.

**Figure 1:**
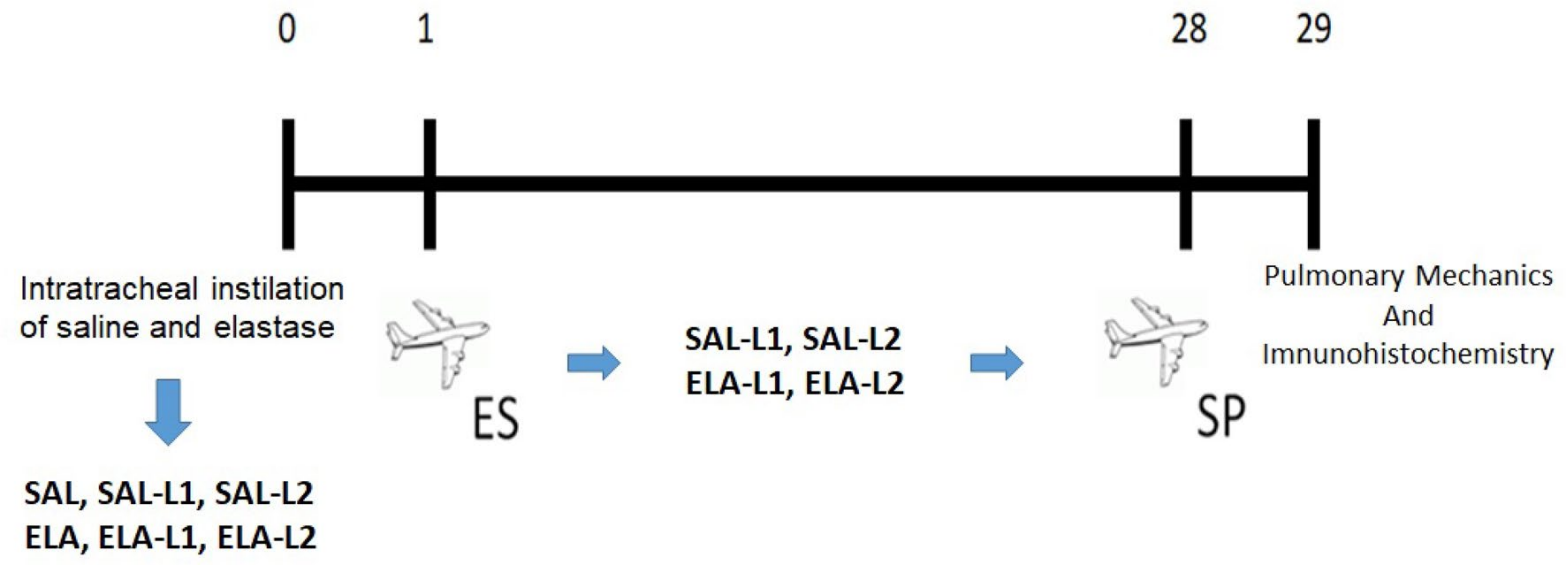
Protocol for elastase-induced emphysema and environmental treatment.

After intratracheal instillation of saline and elastase, the animals were exposed to both locations in the city of Vitória. They remained there for four weeks before returning to the city of São Paulo–SP, Brazil. Pulmonary disease mechanics and pulmonary histopathology were analyzed on the 29th day of the experiment (Fig. 1).

### Environmental exposure

In the environmental exposure groups, the animals from both groups (SAL and ELA) were transported to two locations in the city of Vitória**.** The first location (“Place 1”) is an industrial area where the pelletization of iron ore is carried out. The second location (“Place 2”) was the terrace of a hotel, approximately three miles from Place 1. The local air current directs the pelletizing residues toward Place 2, resulting in the formation of PM in the air.

These locations in the city of Vitória were chosen because of the greater concentration of iron ore dust and due to wind currents in the region, which favors the displacement of this material from Place 1 to Place 2, depending on wind direction.

Due to the climate heterogeneity between seasons in Brazil, it was decided that the experiments would be carried out during two seasons with distinct environmental conditions, including temperature, humidity and wind speed and direction. Therefore, summer and winter were chosen. In the summer, there is an increase in rainfall, temperature and humidity. In the winter, temperatures are milder, precipitation is scarce and humidity levels are extremely low. Due to these climatic factors, it was necessary to send animals during two distinct seasons to evaluate and distinguish the climatic influence on particulate matter. Environmental parameters were obtained from the Vitória meteorological station of the National Institute of Meteorology (INMET).

Particulate matter was continuously collected for 24 h during December 2018 (summer period) and June 2019 to the first week of August 2019 (winter period). Sample collected lasted 18 days, including weekends, in the summer, while sample collection lasted for 36 days in the winter and only included weekdays. During the summer sampling period, the cumulative precipitation was 76.4 mm, the mean temperature was 26 ± 3.5 °C, and the air relative humidity was 74.6 ± 14.4%. During the winter sampling period, the cumulative precipitation was 52.6 mm, the mean temperature was 20.6 ± 3.5 °C, and the air relative humidity was 78.9 ± 16.9%.

The fine (< 2.5 μm) and coarse (2.5–10 μm) fractions of the particulate matter were collected with a dichotomous sampler with a stacked unit filter (SFU) for 47 mm diameter filters described by Hopke et al.^19^. Polycarbonate membrane filters with 8.0 μm and 0.45 μm pore diameters were used to collect the coarse fraction and the fine fraction, respectively. The PM_10_ fraction (< 10 μm) corresponds to the sum of the coarse and fine fractions.

Mass concentrations were obtained gravimetrically by using an electronic high-precision microbalance with 1 μg sensitivity (Mettler Toledo MX5) before and after sampling on polycarbonate membrane filters in a room with controlled temperature and humidity of 22 ± 2 °C and 45 ± 3%, respectively.

Elemental analysis (Al, Si, P, S, Cl, K, Ca, Ti, V, Cr, Mn, Fe, Ni, Zn, Br, and Pb) was performed by EDXRF—Spectrometer EDX 700HS; Shimadzu Corporation^20^. The filter was inputted into the EDXRF instrument, and spectra were accumulated for 900 s under the following conditions: Al filter, vacuum as the X-ray path, 10-mm diameter collimator, 10–20 keV energy range, 50 kV tube voltage, an Rh X-ray tube, and a Si (Li) detector. The spectra were reduced with WinQXAS software, which is available from the website of the International Atomic Energy Agency^21^. Blank filters were also analyzed to remove any false positives in the samples.

### Evaluation of pulmonary mechanics

On Day 29, animals were anesthetized (thiopental sodium, 33 mg/kg i.p.), tracheotomized, and placed in a plethysmograph chamber connected to a small animal ventilator (Harvard Apparatus, South Natick, MA, USA). To abolish their respiratory effort, the animals were given an intraperitoneal injection of pancuronium (0.2 mg/kg). Thereafter, data for oscillatory mechanic calculations were recorded. A signal that generated airflow oscillations at various raw frequencies (0.25 to 19.625 Hz) was used for 16 s, keeping the expiratory valve closed. The generated pressure values were then obtained, and the airway impedance (Pressure/Flow) concerning the various frequencies produced was calculated. A 75% overlapping window was used during the 16 s of sample collection, and three blocks of eight seconds were used to calculate the oscillatory mechanics parameters, according to the equation below:

$${\text{Z}}({\text{f}}) = {\text{Raw}} + {\text{i}}(2\pi {\text{f}}){\text{law}} + \frac{{\left[ {{\text{Gtis}} - {\text{i}}*{\text{Htis}}} \right]}}{{(2\pi {\text{f}})^{\alpha } }}$$

where Z(f) is the impedance of the respiratory system concerning frequency; Raw is the resistance of the airways; i is the imaginary unit (− 1½); f is frequency; Iaw is airway inertance; and α = (2/π)* arctan (Htis/Gtis). And:

$${\text{Ptr}}\left( {\text{t}} \right) = {\text{Ers }} \times {\text{ V}}\left( {\text{t}} \right) + {\text{Rrs }} \times {\text{ V}}^{\prime}\left( {\text{t}} \right),$$

where Ptr is tracheal pressure; t is time; Ers is the elastance of the respiratory system; V is volume; Rrs is the resistance of the respiratory system; and V' is flow. In the ventilatory mechanics calculations, the values were respiratory system resistance (Rrs), respiratory system elastance (Ers), airway resistance (Raw), lung tissue resistance (Gtis), and lung tissue elastance (Htis)^17,18^.

### Bronchoalveolar lavage fluid analysis

Following the evaluation of the respiratory system's mechanics, bronchoalveolar lavage fluid (BALF) was obtained. First, 1.5 mL of saline solution was injected into the tracheal cannula in three 0.5 mL doses. BALF was then centrifuged at 790×*g* for 10 min at 5 °C with an average recovery rate of 80%. The cell pellet was then rehydrated with 300 µL saline solution and vortexed. Subsequently, 100 µL was used to prepare a slide for differential cell counting. The remaining BALF was centrifuged onto a cytospin slide for six minutes at 450 rpm and stained with a Diff-Quick staining kit.

Total cell counts were performed using a Neubauer hemocytometer (400 ×) and light microscopy. Various cell types, such as neutrophils, eosinophils, lymphocytes, and macrophages, were identified using an optical microscope at 1000 × magnification. Approximately 200 cells from each animal were counted^22^.

### Lung histology analysis and immunohistochemistry

We chose to examine additional markers of inflammation, remodeling, and oxidative stress using histology and immunohistochemical analyses.

Following the collection of BALF, the animals were bled, and their hearts and lungs were extracted as a single unit. The lungs were then fixed in 4% formaldehyde at a constant pressure of 20 cmH_2_O for 24 h before being stored in 70% alcohol for up to 36 h before histological processing. Paraffin was used to embed pulmonary tissue fragments.

Five-micrometer sections of the lungs were prepared on slides with (3-aminopropyl) triethoxysilane (Sigma) and processed for histological analysis. To prepare the samples for immunohistochemistry, the sections underwent a series of steps, including deparaffinization, hydration, digestion, and antigen retrieval. The sections were deparaffinized, rehydrated, and treated with proteinase K for 20 min at 37 °C, followed by 20 min at room temperature, before being washed in phosphate-buffered saline (PBS). Endogenous peroxidase was blocked by incubating the sections for three 10-min intervals in 3% hydrogen peroxide (H_2_O_2_).

The slices sections were immunohistochemically stained to evaluate markers such as interleukin-1 beta, 6, 10 and 17 (IL-1β, IL-6, IL-10 and IL-17); tumor necrosis factor alpha (TNF-α); neutrophil elastase; Tissue inhibitor of metalloproteinases 1 (TIMP-1); matrix metallopeptidase 9 and 12 (MMP-9 and MMP-12); transforming growth factor beta (TGF-β); mucin 5 AC (MUC5AC); inducible nitric oxide synthase (iNOS), Gp91phox and nuclear factor kappa B (NFkB). Each section received a dilution of primary antibodies in bovine serum albumin (BSA) solution. Table 1 describes the antibodies, their dilutions, and tags.

**Table 1:** Marker, dilution, primary antibody and specifications.

Marker	Dilution	Primary antibody	Specifications
IL-1β	1:200	Anti-rabbit	SC-7884; Sta Cruz Biotechnology, CA, USA
IL-6	1:100	Anti-rabbit	LS Bio Rabbit; Lifespan C746886
IL-10	1:150	Anti-goat	SC-1783 Goat monoclonal; Sta Cruz Biotechnology, CA, USA
IL-17	1:500	Anti-rabbit	SC-7927 Rabbit Polyclonal; Sta Cruz Biotechnology, CA, USA
Neutrophil elastase	1:500	Anti-rabbit	Abcam Research, AB6872
TIMP-1	1:400	Anti-rabbit	LS-C299465 Mouse Polyclonal; Lifespan Bioscience, Inc., WA, USA
MMP-9	1:600	Anti-rabbit	SC-393859 Mouse Monoclonal; Sta Cruz Biotechnology, CA, USA
MMP-12	1:3000	Anti-rabbit	LS Bio Rabbit; Lifespan C295305
MUCAC	1:500	Anti-mouse	Abcam Research, AB3649
TGF-β	1:400	Anti-rabbit	SC-130348 Mouse Monoclonal; Sta Cruz Biotechnology, CA, USA
iNOS	1:500	Anti-rabbit	SC-7271 Rabbit Polyclonal; Sta Cruz Biotechnology, CA, USA
Gp91phox	1:300	Anti-goat	Sc-53876 Goat Polyclonal; Sta Cruz Biotechnology, CA, USA
NFkB	1:500	Anti-rabbit	SC-8008 Mouse Monoclonal; Sta Cruz Biotechnology, CA, USA

Diluted antibodies were pipetted onto the sections, and the slides were incubated in a humid chamber overnight (18–20 h). On the subsequent day, the slides were rinsed with PBS and subjected to incubation with a secondary antibody from the ABCKit by Vectastain system (Vector Elite-PK-6105 anti-goat, PK-6101 anti-rabbit, and PK-6102 anti-mouse). To visualize immunopositive cells, slides were washed with PBS and proteins were visualized using 3,3′-diaminobenzidine (DAB) chromogen (Sigma Chemical Co., St. Louis, MO, USA). The slides were counterstained with Harris hematoxylin (Merck, Darmstadt, Germany). Finally, the slides were mounted using Entellan microscopy resin (Merck) and subjected to morphometric analysis as described below^23,24^.

Picro–Sirius staining was performed to visualize collagen fibers. The sections were deparaffinized and immersed in water. Samples were subsequently stained with Picro–Sirius solution at room temperature for 1 h, followed by a 5-min wash in running water. Next, the sections were stained with Harris hematoxylin for 6 min and washed in running water for 10 min.

We also performed Perls Prussian blue stain for iron, in which ferric ions (Fe^3+^) are released from cells after treatment with hydrochloric acid and the metal reacts with potassium ferrocyanide to form ferric ferrocyanide, an insoluble bright blue pigment^25^.

### Morphometric analysis

Conventional morphometric analysis was performed using a 100-point, 50-line reticle attached to a microscope eyepiece. The total area of the reticle was 62,500 μm^2^ at 400 × magnification. The dot-counting technique was used to count the alveolar septa by superimposing the reticle onto the peripheral regions of the alveolar septum. For each selected field, the ratio of positive cells to total points on the alveolar septa was calculated, and 10 fields per animal were evaluated at a magnification of 1000 ×^26,27^.

### Mean linear intercept (Lm) measurement

A reticle consisting of 50 straight lines at 200 × magnification was used for microscopic analysis. For each slide, the alveolar septa in the outermost regions were examined in 15 different fields. The reticle was placed on top of the alveolar septa, and the intersections between the lines and the alveolar walls were counted. In this manner, the average diameter of the alveoli was calculated by accounting for the alveolar septum area and the number of intersections between the lines and the septum^15^. The Lm was then calculated using the following formula:

$${\text{Lm }} = { 2}.{5}00\;{\text{m}}/{\text{average}}\;{\text{number}}\;{\text{of}}\;{\text{intersections}}\;{\text{crossing}}\;{\text{the}}\;{\text{alveolar}}\;{\text{walls}}$$

### Image analysis

Collagen content was analyzed using an optical microscope and image analyzer. Images were captured by a DFC 420 camera at 400 × magnification, which was attached to a Leica DM2500 trinocular optical microscope (Leica Microsystems, Wetzlar, Germany), and analyzed using Image-Pro Plus 4.5 software (NIH, Bethesda, MD, USA). To detect fibers, the optical density measurement method was used, and the software provided a threshold for positive areas and quantified them based on the determined area. Ten fields of alveolar septa per animal were examined. The results were expressed as a percentage of the positive area in comparison to the total area^27,28^.

### Evaluation of cytokines in lung tissue

IL-17 level in the bronchoalveolar lavage fluid was quantified using an ELISA kit (Duo Set, R&D Systems, Minneapolis, MN, USA). The immunoassay was performed following manufacturer’s instructions, as previously described by Bittencourt-Mernak et al.^29^.

### Statistical analysis

Scientific Graphing Software SigmaPlot® Version 11.0 was used for all statistical analyses. A one-way analysis of variance (ANOVA) followed by the *Holm‒Sidak* method for multiple comparisons was used to assess differences between groups. These results are expressed as the mean ± standard error of the mean. For correlations between variables, Pearson's correlation was used. For all analyses, p < 0.05 was considered statistically significant.

## Results

### Evaluation of lung mechanics

The examination of lung mechanics has been conducted in numerous animal models of respiratory diseases. In the context of experimental emphysema, a decline in tissue elastance and viscosity has been observed, which is associated with alveolar destruction and the consequent loss of viscoelastic properties. Conversely, the restoration of lung tissue using various components of the extracellular matrix also influences lung function. Modifications in collagen fibers, specifically types I and III, and elastin within the lung parenchyma result in a reduction of lung elasticity.

In the evaluation of lung mechanics in the summer group of mice, there was a significant increase in Rrs in the SAL-L2 group compared to the SAL group (p ≤ 0.05). Similarly, there was an increase in Rrs and Raw in the ELA group compared to the SAL group (p ≤ 0.05). However, there were no differences between groups in the analyses of Ers, Gtis and Htis (Table 2).

**Table 2:** Lung mechanics and positive cell values (± standard error).

Summer						
Lung mechanics	SAL	SAL L1	SAL L2	ELA	ELA L1	ELA L2
Rrs (cmH_2_O s mL^−1^)	0.66 ± 0.02	0.80 ± 0.05	0.97 ± 0.08*	1.01 ± 0.10*	0.76 ± 0.04	0.80 ± 0.01
Ers (cmH_2_O s mL^−1^)	32.58 ± 1.49	35.86 ± 3.52	42.37 ± 2.15	39.73 ± 3.20	37.11 ± 3.65	38.59 ± 1.83
Raw (cmH_2_O s mL^−1^)	0.18 ± 0.02	0.28 ± 0.02	0.42 ± 0.09*	0.50 ± 0.12	0.28 ± 0.01	0.31 ± 0.03
Gits (cmH_2_O s^(1−a)^ mL^−1^)	6.41 ± 0.53	6.80 ± 0.68	8.06 ± 0.29	6.05 ± 0.45	6.67 ± 0.56	6.94 ± 0.31
Hits (cmH_2_O s^(1−a)^ mL^−1^)	33.30 ± 1.91	37.83 ± 3.53	40.71 ± 2.55	36.81 ± 4.79	42.95 ± 2.15	38.43 ± 1.87
Bronchoalveolar lavage fluid (cells × 10^4^/mL)						
Total cells	1.27 ± 0.11	0.79 ± 0.29	1.98 ± 0.50	3.43 ± 0.47*^,#^	2.20 ± 0.35	2.20 ± 0.48
Eosinophils	0.00 ± 0.00	0.00 ± 0.00	0.00 ± 0.00	0.01 ± 0.01	0.04 ± 0.01	0.16 ± 0.14
Neutrophils	0.00 ± 0.00	0.00 ± 0.00	0.15 ± 0.01	0.15 ± 0.15	0.11 ± 0.09	0.02 ± 0.01
Lymphocytes	0.00 ± 0.00	0.01 ± 0.01	0.01 ± 0.00	0.13 ± 0.07	0.02 ± 0.01	0.04 ± 0.01
Macrophages	1.13 ± 0.15	0.87 ± 0.27	1.91 ± 0.49	3.29 ± 0.42*^,#^	2.04 ± 0.30	2.14 ± 0.46
Inflammatory cytokines (cells∕10^4^µm^2^)						
IL-1β	1.79 ± 0.12	8.23 ± 0.55*^,†^	8.33 ± 0.53*^,†^	3.82 ± 0.37*	14.10 ± 0.73*^,†^^,#,&^	13.82 ± 0.77*^,†^^,#,&^
IL-6	4.38 ± 0.68	37.25 ± 0.93*^,†^	36.33 ± 0.87*^,†^	12.21 ± 0.63*	42.27 ± 0.71*^,†^^,#,&^	43.11 ± 0.66*^,†^^,#,&^
IL-10	7.25 ± 0.54	38.89 ± 0.80*^,†^	41.90 ± 0.30*^,†^	15.67 ± 1.18*	48.38 ± 0.42*^,†^^,#,&^	48.37 ± 0.84*^,†^^,#,&^
IL-17	6.52 ± 0.18	25.31 ± 1.93*^,†^	28.04 ± 1.38*^,†^	14.58 ± 1.69*	44.40 ± 1.65*^,†^^,#,&^	44.26 ± 1.48*^,†^^,#,&^
TNF-α	2.02 ± 0.22	44.39 ± 0.60*^,†^	44.82 ± 0.57*^,†^	4.91 ± 0.51*	50.55 ± 0.86*^,†^^,#,&^	50.72 ± 0.83*^,†^^,#,&^
Neutrophil elastase	3.97 ± 0.40	9.67 ± 0.59*^,†^	9.97 ± 1.16*^,†^	6.63 ± 0.61*	13.11 ± 0.53*^,†^^,#,&^	12.63 ± 0.48*^,†^^,#,&^
Oxidative stress (cells∕10^4^µm^2^)						
iNOS	4.53 ± 0.62	32.54 ± 1.88*^,†^	32.81 ± 1.91*^,†^	13.56 ± 1.62*	53.08 ± 3.57*^,†^^,#,&^	53.21 ± 3.95*^,†^^,#,&^
Gp91phox	0.55 ± 0.13	4.11 ± 0.90*^,†^	4,06 ± 1.06*^,†^	0.42 ± 0.16	4.51 ± 0.68*^,†^	4.32 ± 0.65*^,†^
Perls' Prussian blue stains						
Macrophages with iron	0.62 ± 0.16	2.61 ± 0.59*	2.88 ± 0.66*^,†^	0.83 ± 0.27	2.52 ± 0.57*	2.66 ± 0.62*

*p < 0.05 compared to the SAL group; ^†^p < 0.05 compared to the ELA group; ^#^p < 0.05 compared to the SAL-L1 group and ^&^p < 0.05 compared to the SAL-L2 group.

In the winter, there was a significant increase in Raw in the SAL-L2 group compared to that in the SAL, ELA-L1 and ELA-L2 groups (p ≤ 0.05). However, there were no differences in Rrs, Ers, Gtis and Htis between groups (Table 3).

**Table 3:** Lung mechanics and positive cell values (± standard error).

Winter						
Lung mechanics	SAL	SAL L1	SAL L2	ELA	ELA L1	ELA L2
Rrs (cmH_2_O s mL^−1^)	0.66 ± 0.02	0.77 ± 0.07	0.70 ± 0.10	0.83 ± 0.04	0.66 ± 0.05	0.61 ± 0.04
Ers (cmH_2_O s mL^−1^)	32.75 ± 2.32	41.69 ± 3.74	37.91 ± 5.63	34.22 ± 2.23	38.92 ± 4.04	32.29 ± 2.30
Raw (cmH_2_O s mL^−1^)	0.18 ± 0.01	0.23 ± 0.00	0.27 ± 0.030*^,^**	0.20 ± 0.02	0.17 ± 0.01	0.17 ± 0.01
Gits (cmH_2_O s^(1−a)^ mL^−1^)	7.15 ± 0.25	7.42 ± 0.56	6.23 ± 0.51	5.92 ± 0.32	6.42 ± 0.53	6.27 ± 0.33
Hits (cmH_2_O s^(1−a)^ mL^−1^)	28.91 ± 3.23	38.83 ± 2.84	36.88 ± 3.25	39.57 ± 3.36	43.91 ± 3.70	39.30 ± 3.10
Bronchoalveolar lavage fluid (cells × 10^4^/mL)						
Total cells	2.68 ± 0.17	3.03 ± 0.89	2.95 ± 0.68	4.02 ± 0.51	8.10 ± 1.75*^,†^^,#,&^	8.82 ± 1.69*^,†^^,#,&^
Eosinophils	0.00 ± 0.00	0.09 ± 0.04*^,†^^,^**	0.04 ± 0.01	0.00 ± 0.00	0.00 ± 0.00	0.00 ± 0.00
Neutrophils	0.02 ± 0.01	0.17 ± 0.07*^,†^^,^**	0.08 ± 0.01	0.01 ± 0.00	0.00 ± 0.00	0.00 ± 0.00
Lymphocytes	0.00 ± 0.00	0.12 ± 0.04 *^,^**	0.04 ± 0.01	0.05 ± 0.02	0.00 ± 0.00	0.00 ± 0.00
Macrophages	2.65 ± 0.16	2.35 ± 0.76	2.78 ± 0.68	3.96 ± 0.50	8.12 ± 175*^,†^^,#,&^	8.83 ± 1.69*^,†^^,#,&^
Inflammatory cytokines (cells∕10^4^µm^2^)						
IL-1β	1.71 ± 0.17	8.52 ± 0.33*^,†^	8.84 ± 0.78*^,†^	4.02 ± 0.23*	13.68 ± 0.65 *^,†^^,#,&^	14.53 ± 0.69*^,†^^,#,&^
IL-6	4.16 ± 0.64	36.84 ± 1.21*^,†^	35.81 ± 1.11*^,†^	12.63 ± 0.73*	44.00 ± 1.51*^,†^^,#,&^	44.88 ± 1.96*^,†^^,#,&^
IL-10	6.91 ± 0.59	39.77 ± 1.17*^,†^	42.42 ± 0.97*^,†^	15.42 ± 1.15*	47.06 ± 0.79*^,†^^,#,&^	49.81 ± 1.11*^,†^^,#,&^
IL-17	7.22 ± 0.17	22.87 ± 0.58*^,†^	24.88 ± 1.26*^,†^	14.65 ± 0.12*	43.44 ± 2.55*^,†^^,#,&^	41.98 ± 0.57*^,†^^,#,&^
TNF-α	2.19 ± 0.27	44.47 ± 0.49*^,†^	44.09 ± 0.59*^,†^	4.29 ± 0.60*	50.20 ± 0.76*^,†^^,#,&^	51. 35 ± 0.61*^,†^^,#,&^
Neutrophil elastase	3.85 ± 0.25	8.45 ± 0.33*	8.72 ± 1.05*	6.26 ± 0.54*	12.21 ± 0.72*^,†^^,#,&^	12.37 ± 1.03*^,†^^,#,&^
Oxidative stress (cells∕10^4^µm^2^)						
iNOS	5.21 ± 0.87	21.69 ± 2.08*	26.22 ± 1.71*^,†^	14.98 ± 1.33*	50.80 ± 3.92*^,†^^,#,&^	52.44 ± 2.93*^,†^^,#,&^
Gp91phox	0.43 ± 0.15	3.96 ± 0.80*^,†^	4.26 ± 1.18*^,†^	0.52 ± 0.14	4.24 ± 0.63*^,†^	4.09 ± 0.61*^,†^
Perls' Prussian blue stains						
Macrophages with iron	0.72 ± 0.19	3.27 ± 0.74*^,†^	2.83 ± 0.63*	0.89 ± 0.35	3.19 ± 0.61*^,†^	3.35 ± 0.74*^,†^

*p < 0.05 compared to the SAL group; ^†^p < 0.05 compared to the ELA group; ^#^p < 0.05 compared to the SAL-L1 group; ^&^p < 0.05 compared to the SAL-L2 group and **p < 0.05 compared to the ELA-L1 and ELA-L2 groups.

### Cells counts in bronchoalveolar lavage fluid

The number of total cells, eosinophils, neutrophils, lymphocytes, and macrophages in BALF in summer group mice is shown in Table 2 and in winter group mice in Table 3. In the summer group, there were no differences in the number of total cells in BALF between the SAL groups and the ELA groups.

However, there was an increase in the number of total cells in the ELA group compared to the SAL and SAL-L1 groups (p < 0.05). The same pattern was observed in the analysis of macrophages, with no difference in the number of eosinophils, neutrophils, and lymphocytes between groups.

In the winter group, there was an increase in the number of total cells and macrophages in the ELA-L1 and ELA-L2 groups compared to the ELA group and SAL group (p ≤ 0.05). There was an increase in the number of eosinophils, neutrophils, and lymphocytes in the SAL-L1 group compared to the SAL, ELA-L1 and ELA-L2 groups (p ≤ 0.05).

### Mean linear intercept

**Figure 2:**
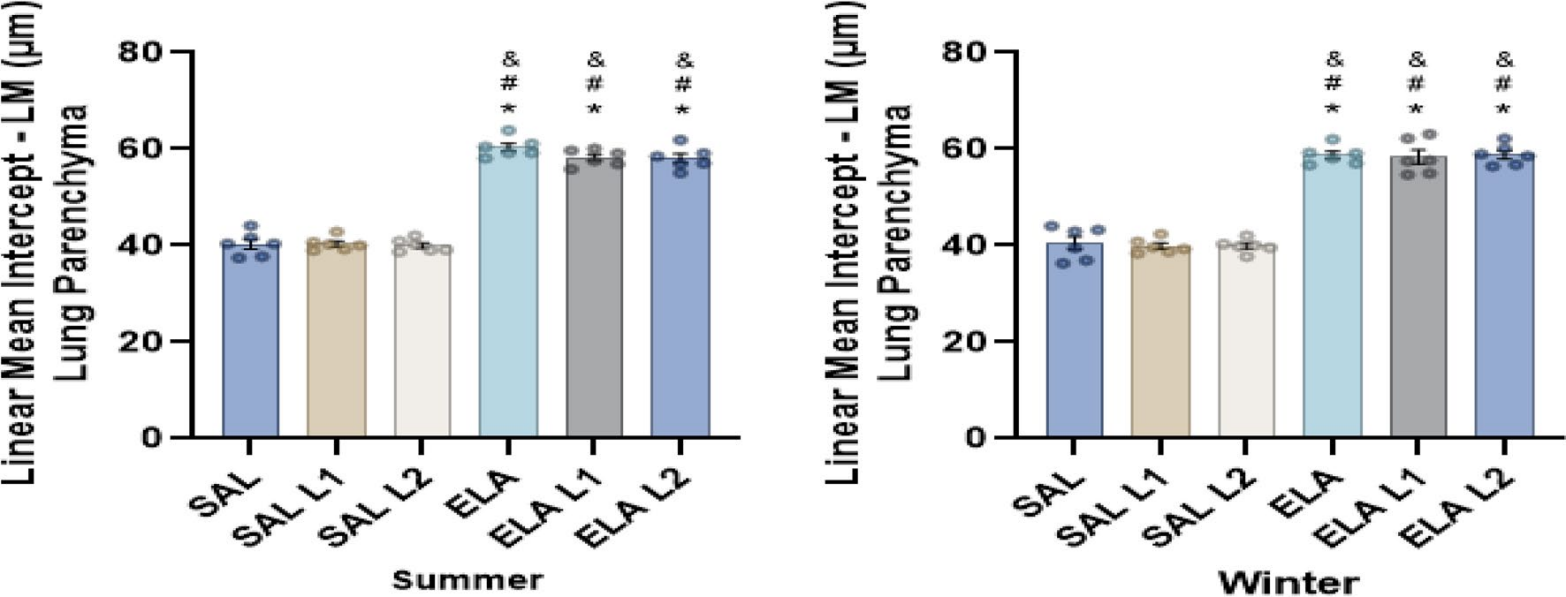
Mean linear intercept (µm^2^ ± standard error): *p < 0.05 when compared to the SAL group; ^#^p < 0.05 when compared to the SAL-L1 group; ^&^p < 0.05 when compared to the SAL-L2 group.

LM is an indicator of the average diameter of the distal air spaces and serves as surrogate measure for the degree of alveolar distention. In both season groups, there were no differences in the Lm within the SAL groups and the ELA groups. However, there was an increase in Lm in the ELA, ELA-L1 and ELA-L2 groups compared to the SAL, SAL-L1 and SAL-L2 groups, respectively (p ≤ 0.05) (Fig. 2).

### Inflammatory cytokines

In both the summer and winter mice,there was an increase in the number of positive cells of inflammatory cytokines in the ELA, SAL-L1, ELA-L1, SAL-L2 and ELA-L2 groups compared to the SAL group (p ≤ 0.05) (Tables 2, 3).

In addition, there was also an increase in the number of positive cells of all markers the SAL-L1, ELA-L1, SAL-L2 and ELA-L2 groups compared to the ELA group (p ≤ 0.05). Last, there was an increase in all markers in the ELA-L1 and ELA-L2 groups compared to the SAL-L1 and SAL-L2 groups (p ≤ 0.05).

### Extracellular matrix remodeling markers

**Figure 3:**
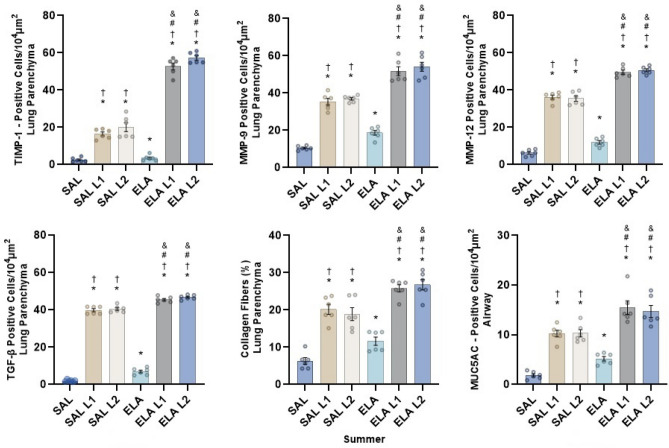
Remodeling markers in summer (TIMP-1, MMP-9, MMP-12, MUC5AC, and TGF-β) positive cell values (cells/10^4^ µm^2^ ± Standard Error): *p < 0.05 compared to the SAL group; ^†^p < 0.05 compared to the ELA group; ^#^p < 0.05 compared to the SAL-L1 group and ^&^p < 0.05 compared to the SAL-L2 group.

In the summer group (Fig. 3), there was an increase in TIMP-1, MMP-9, MMP-12, TGF-β, collagen fibers and MUC5AC in the SAL-L1 and SAL-L2 groups compared to the SAL group, as well as in the ELA-L1 and ELA-L2 groups compared to the ELA group (p ≤ 0.05). When all groups were compared, there was an increase in the number of positive cells of remodeling markers in the SAL-L1, ELA-L1, SAL-L2 and ELA-L2 groups compared to the SAL and ELA groups (p ≤ 0.05).

**Figure 4:**
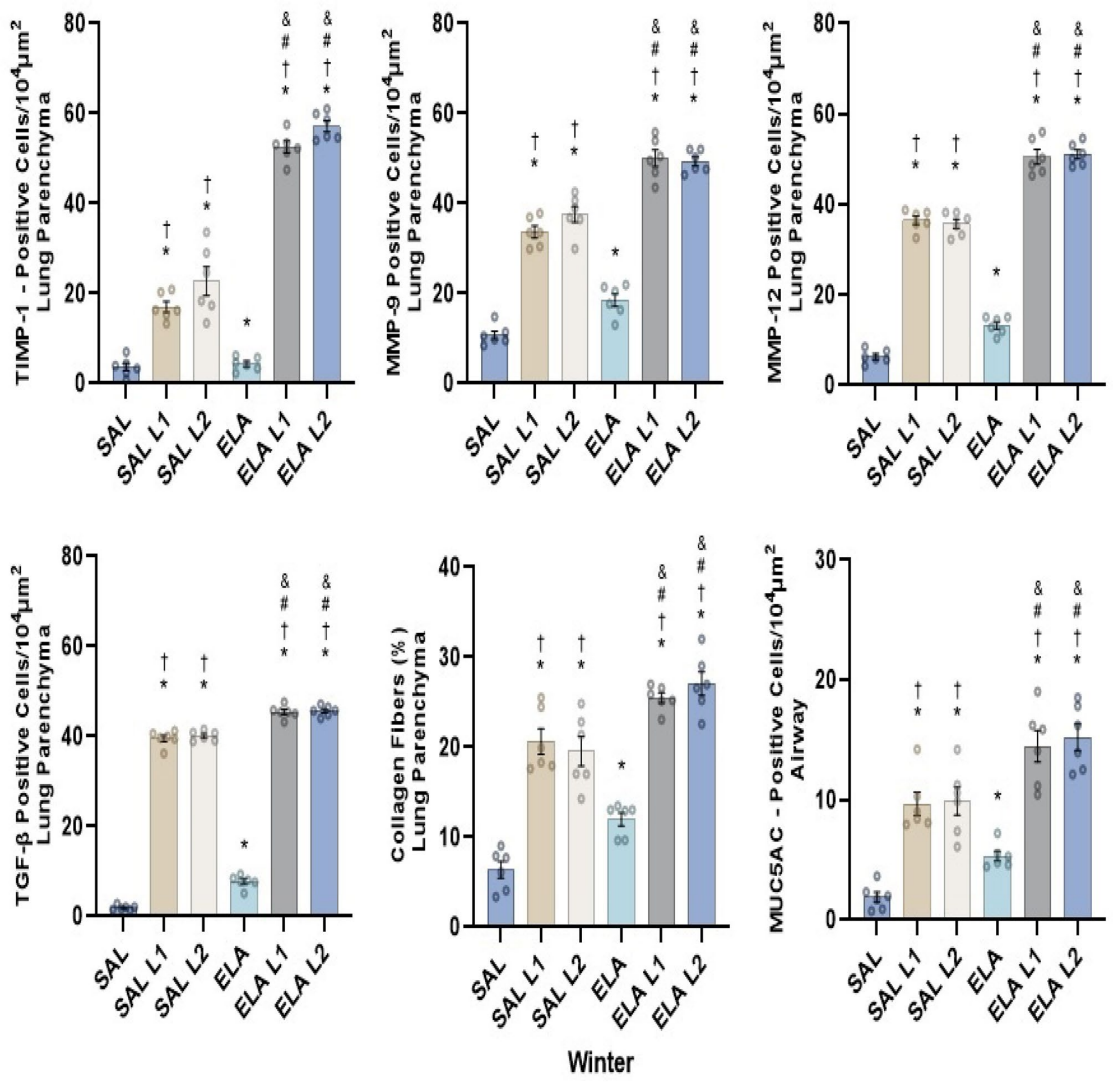
Remodeling markers in winter (TIMP-1, MMP-9, MMP-12, MUC5AC, and TGF-β) positive cell values (cells/10^4^ µm^2^ ± standard error): *p < 0.05 when compared to the SAL group; ^†^p < 0.05 when compared to the ELA group; ^#^p < 0.05 when compared to the SAL-L1 group and ^&^p < 0.05 when compared to the SAL-L2 group.

Collagen fibers, MMP-9, MMP-12, TGF-β, and MUC5AC also increased in the ELA group compared to the SAL group (p ≤ 0.05). Furthermore, there was an increase in all markers in the ELA-L1 and ELA-L2 groups compared to the SAL-L1 and SAL-L2 groups (p ≤ 0.05). The results of the winter mice were similar to those of the summer mice (Fig. 4).

### Oxidative stress markers

In the summer mice (Table 2), there was an increase in the number of iNOS-positive cells in the SAL-L1 and SAL-L2 groups compared to the SAL group (p ≤ 0.05), as well as in the ELA-L1 and ELA-L2 groups compared to the ELA group (p ≤ 0.05). When all groups were compared, there was an increase in the ELA, SAL-L1, ELA-L1, SAL-L2, and ELA-L2 groups compared to the SAL group (p ≤ 0.05).

There was also an increase in the SAL-L1, ELA-L1, SAL-L2, and ELA-L2 groups compared to the ELA group (p ≤ 0.05). Furthermore, there was an increase in the ELA-L1 and ELA-L2 groups compared to the SAL-L1 and SAL-L2 groups (p ≤ 0.05). The results of the winter mice were similar, except that there were no differences in number of iNOS positive cells between the SAL-L1 and ELA groups (Table 3).

In both the summer and winter mice (Tables 2, 3), there was an increase in the number of Gp91phox-positive cells in the SAL-L1 and SAL-L2 groups compared to the SAL group (p ≤ 0.05), as well as in the ELA-L1 and ELA-L2 groups compared to the ELA group (p ≤ 0.05). When all groups were compared, there was an increase in the SAL-L1, ELA-L1, SAL-L2, and ELA-L2 groups compared to the SAL and ELA groups (p ≤ 0.05).

### Mechanisms involved (NFkB)

**Figure 5:**
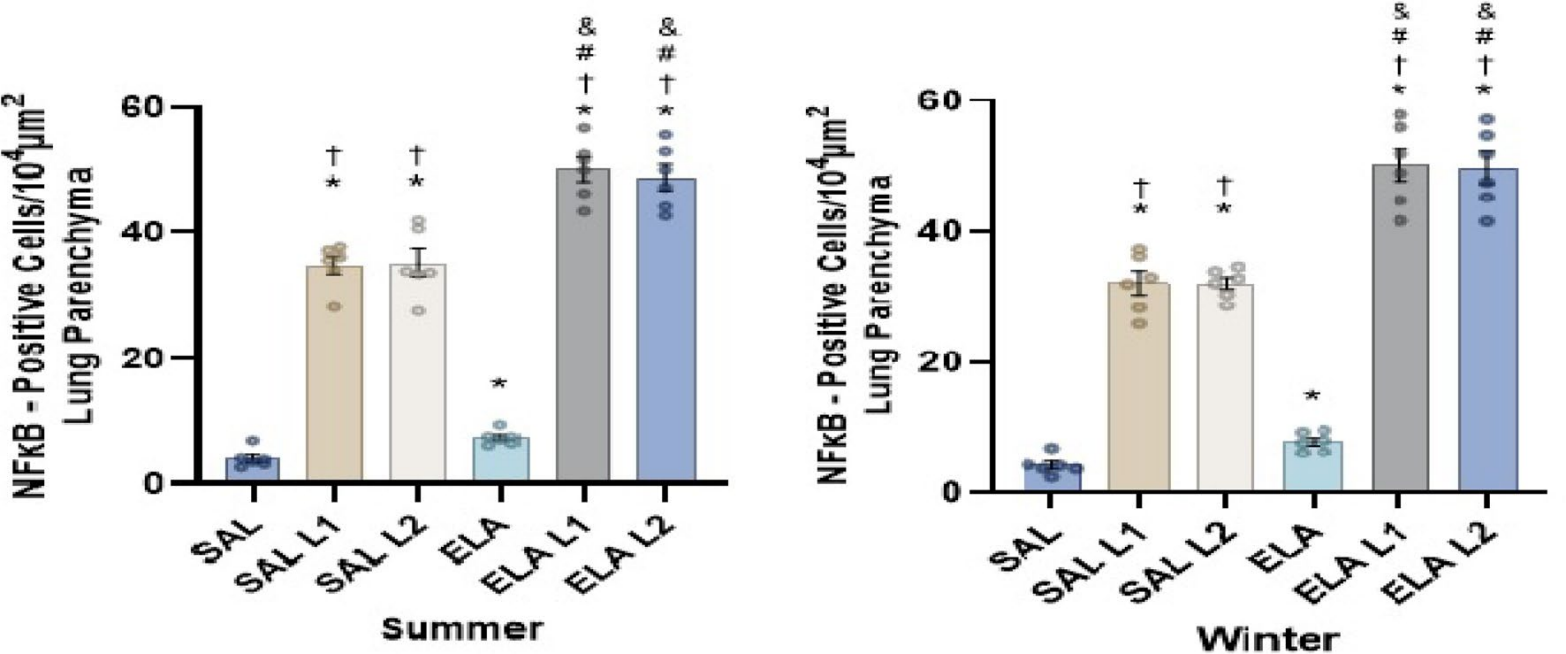
NFkB positive cell values (cells/10^4^ µm^2^ ± standard error): *p < 0.05 when compared to the SAL group; ^†^p < 0.05 when compared to the ELA group; ^#^p < 0.05 when compared to the SAL-L1 group and ^&^p < 0.05 when compared to the SAL-L2 group.

In both the summer and winter mice (Fig. 5), there was an increase in the number of cells positive for NFkB in the SAL-L1 and SAL-L2 groups compared to the SAL group (p < 0.05). There was also an increase in the number of NFkB positive cells in the ELA, ELA-L1, and ELA-L2 groups compared to the SAL group (p < 0.05).

In addition, we observed an increase in the number of NFkB positive cells in the ELA-L1 and ELA-L2 groups compared to the ELA group (p < 0.05). The number of positive cells also increased in the SAL-L1 and SAL-L2 groups compared to the ELA group (p < 0.05), as well as in the ELA-L1 and ELA-L2 groups compared to the SAL-L1 and SAL-L2 groups (p < 0.05).

### Perls prussian blue stains of lung tissue: macrophages with iron

In the summer mice (Table 2), there was an increase in the number of macrophage with iron in the SAL-L1 and SAL-L2 groups compared to the SAL group (p ≤ 0.05). We did not observe any differences in the ELA, ELA-L1 and ELA-L2 groups, but we observed differences in the ELA-L1 and ELA-L2 compared to the SAL group (p ≤ 0.05).

In the winter mice (Table 3), there was an increase in the number of macrophage with iron in the SAL-L1 and SAL-L2 groups compared to the SAL group (p ≤ 0.05), as well as in the ELA-L1 and ELA-L2 groups compared to the SAL and the ELA groups (p ≤ 0.05).

### Pearson's correlation

In the summer mice, there were low to moderate correlations between lung tissue elastance and resistance (Gtis and Htis) and the following markers: IL-1β, IL-6, IL-10, IL-17, TNF-α, neutrophil elastase, TIMP-1, MMP-9, MMP-12, TGF-β and Gp91phox. There was also a moderate correlation between Gtis and the following markers: collagen fibers, iNOS and NFkB (Table 4).

**Table 4:** Pearson correlation between Gtis and Hits with inflammation, remodeling, oxidative stress and mechanisms involved markers.

	Summer				Winter	
	Gtis		Hits		Hits	
	Correlation coefficient	P value	Correlation coefficient	P value	Correlation coefficient	P value
Inflammatory markers						
IL-1β	0.465	0.015*	0.447	0.006*	0.379	0.023*
IL-6	0.465	0.004*	0.386	0.023*	0.395	0.017*
IL-10	0.484	0.003*	0.435	0.008*	0.360	0.031*
IL-17	0.434	0.008*	0.349	0.037*	0.422	0.010*
TNF-α	0.500	0.002*	0.386	0.020*	0.331	0.049
Neutrophil elastase	0.545	0.001*	0.366	0.028*	0.496	0.002*
Remodeling markers						
TIMP-1	0.458	0.005*	0.436	0.008*	0.383	0.021*
MMP-9	0.476	0.003*	0.416	0.010*	0.433	0.008*
MMP-12	0.481	0.003*	0.421	0.010*	0.407	0.014*
TGF-β	0.496	0.002*	0.387	0.020*	0.394	0.017*
Collagen fibers	0.472	0.003*	0.293	0.08	0.422	0.010*
Oxidative stress						
iNOS	0.402	0.015*	0.386	0.017*	0.435	0.008*
Gp91phox	0.466	0.004*	0.341	0.041*	0.262	0.122
Mechanisms involved						
NFκB	0.477	0.003*	0.411	0.013*	0.381	0.021*

*p < 0.05.

In the winter mice, there was a correlation between Htis and the following markers: IL-1β, IL-6, IL-10, IL-17, TNF-α, neutrophil elastase, TIMP-1, MMP-9, MMP-12, TGF-β, collagen fibers, iNOS and NFkB (Table 4).

In both the summer and winter mice, there were moderate correlations macrophages with iron and the following markers: IL-1β, IL-6, IL-10, IL-17, TNF-α, neutrophil elastase, TIMP-1, MMP-9, MMP-12, TGF-β, collagen fibers, iNOS Gp91phox and NFkB (Table 5).

**Table 5:** Pearson correlation between perls with inflammatory, remodeling, oxidative stress and mechanisms involved markers.

	Perls Prussian blue stains: macrophages with iron			
	Summer		Winter	
	Correlation coefficient	P value	Correlation coefficient	P value
Inflammatory markers				
IL-1β	0.539	0.001*	0.610	0.001*
IL-6	0.614	0.001*	0.648	0.001*
IL-10	0.597	0.001*	0.599	0.001*
IL-17	0.508	0.001*	0.549	0.001*
TNF-α	0.612	0.001*	0.650	0.001*
Neutrophil elastase	0.551	0.001*	0.523	0.001*
Remodeling markers				
TIMP-1	0.417	0.011*	0.486	0.002*
MMP-9	0.491	0.002*	0.588	0.001*
MMP-12	0.549	0.001*	0.633	0.001*
TGF-β	0.615	0.001*	0.640	0.001*
Collagen fibers	0.553	0.001*	0.504	0.001*
Oxidative stress				
iNOS	0.502	0.001*	0.479	0.003*
Gp91phox	0.348	0.037*	0.565	0.001*
Mechanisms involved				
NFκB	0.502	0.001*	0.629	0.001*

*p < 0.05.

### Evaluation of cytokines in lung tissue

In both the summer and winter mice, there was an increase in the level of IL-17 in the SAL-L1 and SAL-L2 groups compared to the SAL group (p < 0.05). There was also an increase in the level of IL-17 in the ELA, ELA-L1, and ELA-L2 groups compared to the SAL group (p < 0.05). In addition, we observed an increase in the level of IL-17 in the ELA-L1 and ELA-L2 groups compared to the ELA group (p < 0.05). The level of IL-17 also increased in the SAL-L1 and SAL-L2 groups compared to the ELA group (p < 0.05).

**Figure 6:**
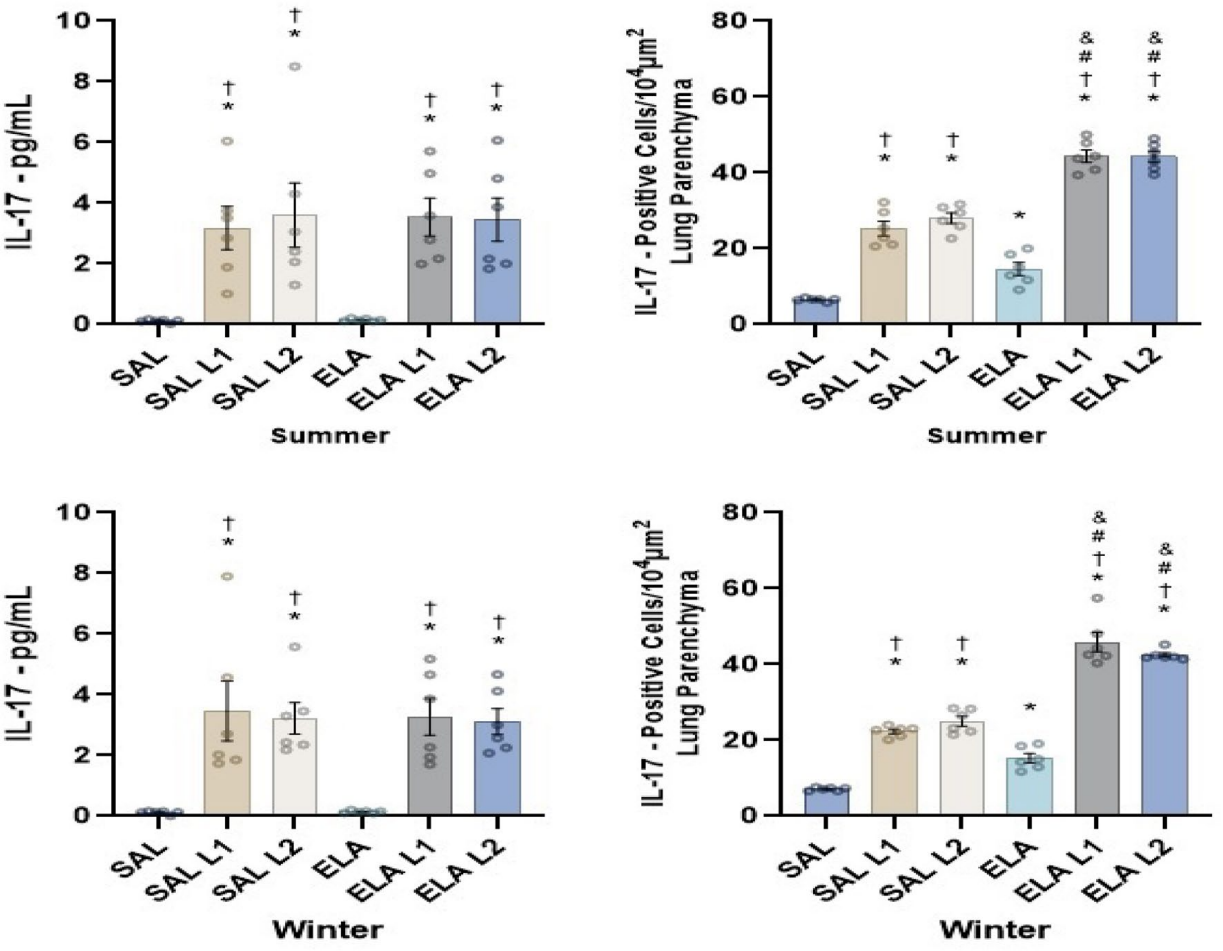
Level of IL-17 (pg/mL ± standard error) measured by ELISA and results of positive cell expression assessed by immunohistochemistry for cell-associated cytokine production of IL-17 (cells/10^4^ µm^2^ ± standard error) in lung parenchyma. *p < 0.05 when compared to the SAL group; ^†^p < 0.05 when compared to the ELA group; ^#^p < 0.05 when compared to the SAL-L1 group and ^&^p < 0.05 when compared to the SAL-L2 group.

Despite the results of the Level of IL-17 measured by ELISA through bronchoalveolar lavage, these findings closely resemble the results of positive cell expression assessed by immunohistochemistry for cell-associated cytokine production of IL-17 in lung parenchyma (Fig. 6), demonstrating a moderate correlation between ELISA and immunohistochemistry results for IL-17 in both summer (correlation coefficient = 0.624; p = 0.0001) and winter (correlation coefficient = 0.603; p = 0.0001).

### Qualitative analysis

**Figure 7:**
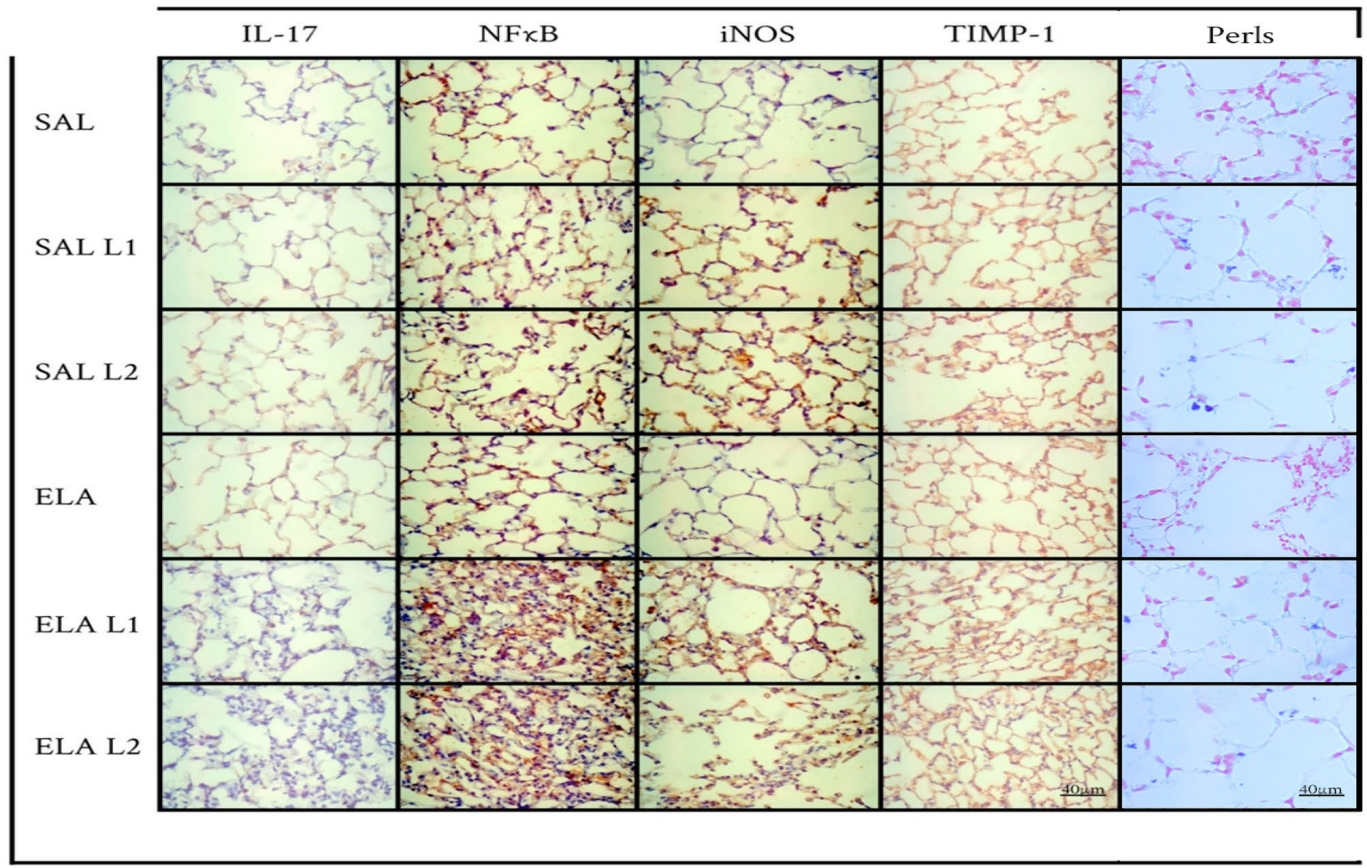
Inflammatory, mechanism involved, oxidative stress, remodeling markers and macrophages with iron: Photomicrographs of IL-17, NFkB, iNOS, TIMP-1 and Perls. Immunohistochemical staining in the airways. 400 × magnification. All experimental groups are represented as SAL, SAL-L1, SAL-L2, ELA, ELA-L1, and ELA-L2 in summer.

**Figure 8:**
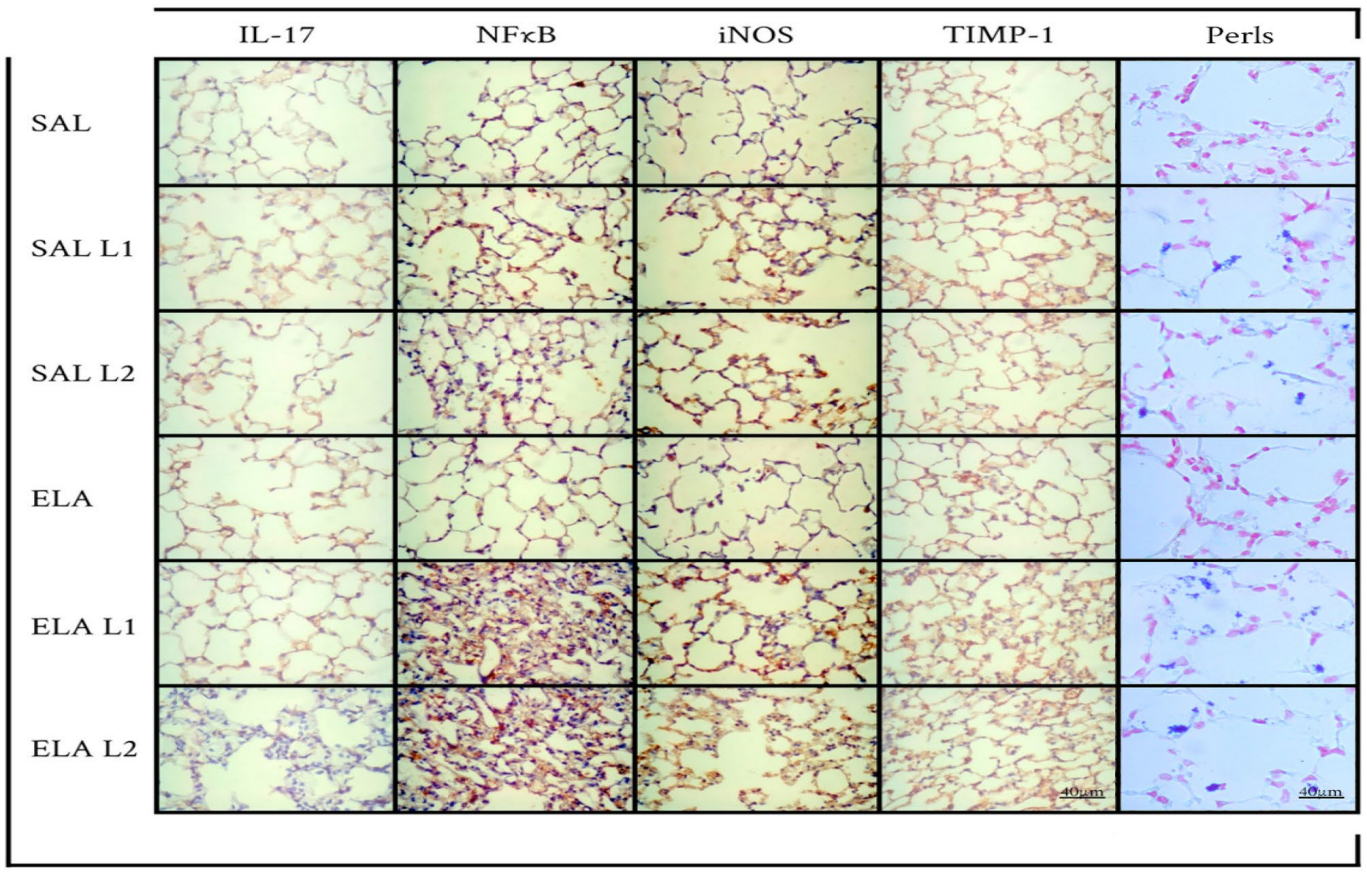
Inflammatory, mechanism involved, oxidative stress, remodeling markers and macrophages with iron: Photomicrographs of IL-17, NFkB, iNOS, TIMP-1 and Perls. Immunohistochemical staining in the airways. 400 × magnification. All experimental groups are represented: SAL, SAL-L1, SAL-L2, ELA, ELA-L1, and ELA-L2, in winter.

Representative photomicrographs (shown in Figs. 7 and 8 for the summer group and winter group, respectively) were taken to illustrate the inflammatory processes, mechanism involved, extracellular matrix remodeling, oxidative stress and macrophages with iron. IL-17 was the markers for inflammation; Inflammation, as indicated by markers such as IL-17, represents the immune response in lung tissue. It involves the activation of immune cells and release of inflammatory mediators, which can lead to tissue damage and dysfunction. NFkB was the markers for the mechanism involved; NFκB, a transcription factor, plays a key role in regulating the expression of genes involved in inflammation and immune responses. Activation of NFκB signaling pathways can lead to increased production of inflammatory cytokines and chemokines, exacerbating inflammation in lung tissue. TIMP-1 was the marker for the extracellular matrix remodeling; Remodeling, assessed through markers such as TIMP-1, refers to the structural changes in lung tissue. This includes alterations in the extracellular matrix, such as collagen deposition and elastin breakdown, leading to changes in lung architecture and function. iNOS was the marker for the oxidative stress; Oxidative stress, identified by markers like iNOS, occurs when there is an imbalance between the production of reactive oxygen species (ROS) and the body's ability to neutralize them with antioxidants. In the lung, oxidative stress can cause damage to cells and tissues, contributing to inflammation and respiratory diseases. Perls was the markes for the macrophages with iron; Accumulation of macrophages, identified by Perls staining for macrophages containing iron, signifies the presence of these immune cells in the lung tissue.

### The concentration of particulate matter

The concentration of PM found in the air of the city of Vitória and the elements present in this PM are represented in Tables 6 and 7.

**Table 6:** Atmospheric PM concentrations (µg.m^-3^) collected at each sampling station and during the summer and winter seasons.

	Summer	(December 2018)		Winter	(June–August 2019)	
	Coarse	Fine	PM_10_	Coarse	Fine	PM_10_
Place 1 (µg m^−3^)						
N	18	18	18	36	36	36
Mean	145	18.6	164	41.7	9.56	51.2
Std	112	8.98	112	24.8	4.80	27.8
Min	19.7	6.27	26.0	15.1	2.43	17.6
Max	378	35.4	386	103	23.0	114
Median	116	17.6	136	31.3	8.55	39.6
Place 2 (µg m^−3^)						
N	18	18	18	36	36	36
Mean	29.6	4.02	33.6	28.9	7.71	36.6
Std	11.4	1.80	12.1	11.2	3.74	13.0
Min	12.1	1.47	14.5	11.6	3.11	15.1
Max	53.2	7.39	57.1	57.0	20.5	73.0
Median	27.1	3.49	32.4	25.6	6.84	33.0

**Table 7:** Elemental concentrations in the fine and coarse PM fractions at both the sampling station and sampling period.

	Summer December 2018					Winter June–July 2019				
	n	Mean	Std	Min	Max	n	Mean	Std	Min	Max
		(µg m^−3^)					(µg m^−3^)			
Place 1										
Coarse PM										
Cl	16	2.260	1.255	0.625	5.309	28	2.146	1.739	0.357	6.632
Fe	16	40.55	33.74	2.910	116.2	28	9.931	8.049	1.947	31.72
Na	12	0.584	0.590	0.044	1.686	27	0.393	0.324	0.050	1.252
S	16	1.241	0.828	0.420	3.014	28	0.396	0.207	0.167	0.875
Fine PM										
Cl	16	0.227	0.253	0.012	0.796	27	0.191	0.182	0.022	0.628
Fe	16	5.504	3.544	0.598	12.62	27	1.691	1.456	0.056	5.880
Na	12	0.323	0.324	0.029	0.925	20	0.106	0.091	0.010	0.342
S	16	1.319	0.653	0.328	2.234	27	0.361	0.259	0.056	1.124
Place 2										
Coarse PM										
Cl	18	5.933	1.606	2.703	9.042	18	4.530	1.461	2.448	7.128
Fe	18	3,.35	3.001	0.260	9.341	18	1.209	0.871	0.217	3.361
Na	18	1.336	0.354	0.665	2.350	18	0.907	0.278	0.459	1.484
S	18	0.812	0.257	0.373	1.396	18	0.601	0.166	0.366	0.907
Fine PM										
Cl	17	0.160	0.187	0.004	0.746	18	0.213	0.130	0.035	0.486
Fe	17	0.172	0.208	0.022	0.749	18	0.354	0.345	0.042	1.146
Na	14	0.131	0.096	0.023	0.410	18	0.119	0.078	0.010	0.267
S	17	0.324	0.303	0.032	1.288	18	0.350	0.224	0.088	0.941

*n* number, *Std.* standard deviation, *Min.* minimum value, *Max.* maximum value.

Table 6 indicates that the predominant composition in the atmosphere was PM_10_, highlighting its prevalence in the sampled air. Additionally, Table 7 reveals the presence of various elements beyond iron, including chlorine (Cl), sulfur (S), and sodium (Na). These findings suggest a complex composition of atmospheric pollutants, potentially contributing to adverse health effects. Elements such as Cl, S, Na, alongside iron, have been associated with increased inflammation, tissue remodeling, and oxidative stress when present in the atmosphere. This underscores the importance of understanding the composition of atmospheric pollutants and their potential implications for respiratory health.

## Discussion

This study demonstrates that environmental exposure significantly promoted in inflammation, oxidative stress, and remodeling in healthy animals by potentially acting on NFkB. Moreover, it is interesting that these changes were similar to those observed in animals with elastase-induced emphysema that were not exposed to environmental exposure to iron powder.

Only the local pollution from Place 1 induced an inflammatory response, and this response was exacerbated when it was associated in animals that already had an inflammatory condition induced by emphysema.

A geochemical study of the region was performed during the different seasons to characterize the particulate material. Atmospheric particulate matter was sampled at the same location during the summer and winter seasons. In both summer and winter, the mean PM concentrations at Place 1 were higher than those at Place 2. During the summer period, the PM_10_ concentrations were 164 ± 112 μg m^−3^ and 33.6 ± 12.1 μg m^−3^ at Place 1 and Place 2, respectively. During the winter period, the PM_10_ concentrations were 51.2 ± 27.8 μg m^−3^ and 36.6 ± 13.0 μg m^−3^ at Place 1 and Place 2, respectively. The results also showed that the coarse fraction represented approximately 80% of PM_10,_ and for both collection sites and seasons, coarse PM concentrations were higher than fine PM concentrations.

The PM_2.5_ concentrations obtained in this study were lower than the mean values obtained by Miranda et al. (2012) in Brazilian cities such as São Paulo (28.1 ± 13.6 µg m^−3^), Rio de Janeiro (17.2 ± 11.2 µg m^−3^), Belo Horizonte (14.7 ± 7.7 µg m^−3^), Curitiba (14.4 ± 9.5 µg m^−3^) and Porto Alegre (13.4 ± 9.9 µg m^−3^).

Furthermore, in both summer and winter, the mean concentrations at Place 1 were higher than those at Place 2. PM enters the human body through inhalation, and prolonged exposure to PM can worsen lung inflammation due to its direct toxic effects and production of oxidative stress^2^.

It should also be noted that, in addition to the PM, chemical composition is a key determinant of the inflammatory response^5^. Considering the particulate matter elemental composition, we have selected chlorine (Cl), iron (Fe), sodium (Na) and sulfur (S) for discussion since they showed the highest concentrations. In the winter, chlorine concentrations were higher at Place 2 than at Place 1 in both coarse and fine PM fractions.

Increased exposure to sulfur dioxide (SO_2_) and nitrogen dioxide (NO_2_) at World Health Organization-acceptable concentrations, as well as PM less than or equal to 10 µm in aerodynamic diameter, have been linked to an increase in mortality in COPD patients^30^.

There was no difference in lung mechanics among the groups in either summer or winter mice. Nonetheless, there were low to moderate correlations between lung tissue elastance and resistance and indicators of inflammation, oxidative stress, and remodeling. Hantos et al. discovered that mice given an intratracheal injection of elastase had an increase in volume and a decrease in lung tissue elastance but no change in airway and lung tissue resistance, suggesting that lung tissue destruction is not always linked to lung system dysfunction^31^.

Mice exposed to fine particulate matter inhalation for four hours showed a slight but not significant increase in respiratory elastance and resistance^32^. There were no changes in lung function after two weeks of exposure to PM in a high-concentration environment; changes in lung function occurred only after four weeks of exposure^33^.

It should be noted that the animals in this study were exposed to ambient air in three different locations for four weeks, leading to the hypothesis that the exposure time was insufficient to cause changes in pulmonary mechanics.

The presence of chlorine in the atmospheric PM in Vitoria may have influenced the inflammatory response and remodeling. Exposure to elevated levels of chlorine in the environment is associated with an increased occurrence of lung inflammation due to chlorine's ability to react with respiratory mucous membranes and trigger inflammatory responses in the respiratory system. De Genaro et al. discovered that both acute and chronic exposure to chlorine gas reduces lung function and increases oxidative stress and mucus secretion in healthy mice^26^. When inhaled, chlorine can become solubilized in the bronchoalveolar fluid, cross the cell membrane, react with local proteins, activate local inflammation, and cause epithelial damage due to oxidative stress^34–36^.

When a proinflammatory response is activated, reactive oxygen species (ROS) and proinflammatory cytokines such as TNF-α, IL-1β and interferon-gamma (INF-γ) are released, which activate iNOS^37,38^. At the site of inflammation, this enzyme produces nitric oxide (NO), which increases oxidative stress^39^. Proinflammatory cytokines can activate the Th17 response, resulting in the production of IL-17 and the recruitment of neutrophils, as well as tissue remodeling and mucus production^40^.

A single exposure to low doses of chlorine potentiated the Th2 response in asthmatic mice, resulting in increased inflammation, altered lung function, and activation of iNOS and kinase 2 (ROCK-2) signaling. Similar responses were observed in healthy animals exposed to low concentrations of chlorine^41^.

When compared to the animals kept in a vivarium, those exposed in Vitória showed an increase in iNOS. The cytogenotoxic action of PM may be directly linked to oxidative stress. Several studies have been conducted to determine the cytogenotoxic action of PM^42^, which has been primarily attributed to metallic components bound or adsorbed on particles, particularly transition metals capable of inducing the formation of reactive oxygen species (ROS), such as iron^43^.

Additionally, the presence of chlorine in the atmosphere can exacerbate this cytogenotoxic action. Chlorine, when combined with certain metallic components in PM, can lead to the formation of highly reactive chlorine radicals and further enhance oxidative stress in cells. This combined action of metallic components and chlorine can contribute to the development of respiratory inflammation and other health effects in individuals exposed to polluted air^44^.

Through the Haber–Weiss and Fenton reactions, iron particles stimulate the production of hydroxyl radicals, which causes oxidative stress in cells^43,45^. According to research, reactive oxygen species (ROS) can be produced on the surface of particles as a result of the absorption of polycyclic aromatic hydrocarbons (PAHs) and nitro-PAHs. The Fenton reaction, which is catalyzed by transition metals such as iron, copper, chromium, and vanadium, produces the highly reactive hydroxyl radical by combining Fe^2+^, H_2_O_2_, and H^+^, which can cause oxidative damage in DNA^45^.

When compared to the animals that remained in a vivarium, the animals exposed in Vitória showed a significant increase in the in the number of positive cells of all remodeling markers. Long-term chronic exposure to PM resulted in impaired lung function, emphysematous lesions, airway inflammation, and airway wall remodeling. Exposure to PM significantly increases the expression of MMP9, MMP12, fibronectin, collagen, and TGF-β1 proteins, regardless of concentration^41^. Evidence has revealed that several PM components can cause cellular harm, which in turn can activate pathways for extracellular matrix remodeling^46,47^. Airway remodeling refers to structural and extracellular matrix (ECM) changes in large and small airways^48^. Previous studies have reported that the ECM of airway cells is altered in asthmatic patients, with a decrease in type IV collagen and elastin levels and an increase in type I collagen, fibronectin, laminin, periostin, versican, and decorin levels and lumican deposition^48,49^.

Proinflammatory factors such as cytokines and proteases are secreted, which further triggers immune responses and contributes to ECM remodeling^39,40^. Numerous immune cells, including but not limited to neutrophils, eosinophils, monocytes, macrophages, and mast cells, play a role in this process^50^.

Even though PM_10_ is present in higher concentrations in the air of Vitória-ES, particles with diameters smaller than 2.5 μm can penetrate the bronchioles and alveoli, making it the most dangerous particle type for the lungs^41^. These particles can remain in the atmosphere for a longer period, increasing the likelihood of inhalation and the rate at which the composition of the air changes. The health consequences range from an increased risk of cardiovascular disease, chronic lung inflammation, and decreased lung function to an increase in asthma attacks^10^.

Chan et al. discovered an increase in lymphocytes and macrophages, which was also observed in our mouse group exposed to high doses of PM. Nonetheless, exposure to 5 μg of PM_10_ did not result in the activation of eosinophil- or neutrophil-driven inflammation. As expected, the increase in IL-1β levels was linked to the activation of the NLRP3 inflammasome^42^. In another study, daily exposure to 50 µg of PM_2.5_ for three weeks increased both IL-1β and TGF-β1 levels in bronchoalveolar lavage fluid^51^.

According to Chu et al., PM_2.5_ inhalation can exacerbate macrophage-induced damage in the air sacs of mice with COPD. They discovered that IL-6, IL-8, and TNF-α levels increased in bronchoalveolar lavage fluid, exacerbating airway inflammation. Researchers have concluded that PM_2.5_ can upregulate the expression of genes encoding TNF-α, IL-6, and IL-1β^52^.

Increased exposure to PM_2.5_ can cause goblet cell hyperplasia and excessive mucus secretion in mice with COPD by increasing the expression levels of MUC5AC, MUC5B, collagen I, and collagen III in lung tissue^33^. MUC5AC levels increased in mice at both exposure locations, both in the SAL-L1 and SAL-L2 control groups and in the ELA-L1 and ELA-L2 elastase groups, with MUC5AC levels being higher in the ELA-L1 and ELA-L2 groups than in the other groups.

Wang et al. discovered that PM_2.5_ has a substantial impact on exacerbating COPD symptoms. According to the findings, PM_2.5_ causes increased oxidative stress, airway inflammation, and goblet cell hyperplasia, which leads to imbalanced protease/antiprotease levels and airway remodeling. PM_2.5_ deposited in the pulmonary bronchioles and alveoli causes oxidative stress, which initiates a chain reaction of harmful processes such as protease activation and increased bronchial inflammation, resulting in increased mucus hypersecretion, small airway fibrosis, and collagen accumulation^42^.

As a result, there is persistent inflammation and the development of pulmonary emphysema^42^. Feng et al. discovered that mice exposed to high levels of PM_2.5_ for four weeks had poor lung function, mucus hypersecretion, and high levels of proinflammatory cytokines and oxidative stress indicators. According to the authors, four weeks may be sufficient time to achieve the histological changes caused by PM inhalation^33^.

We observed an increase in iron deposition in alveolar macrophages in healthy, elastase-exposed mice. Although we did not observe a correlation with this iron deposition and functional changes. We noted a moderate correlation between inflammation, remodeling, oxidative stress and NFkB with the number of iron-positive macrophages. Seaton et al. demonstrated that dust in the London Underground had cytotoxic and inflammatory potential at high doses, which was consistent with the iron oxide found in the dust^53^. The presence of soluble metals, such as iron, nickel, vanadium, cobalt, copper, and chromium, in inhaled particles may cause an increase in cellular oxidative stress in airway epithelial cells^54^.

Some free radicals generated from oxidative stress have been shown to activate specific protein transcription factors, including NFkB, which upregulates the expression of genes for cytokines, chemokines, and other inflammatory mediators, as well as apoptosis- and necrosis- related genes in macrophages and respiratory epithelial cells, impairing immune defense processes and increasing airway reactivity^55,56^. In this study, NFkB levels were higher in the SAL-L1, SAL-L2, ELA-L1, and ELA-L2 groups than in the SAL and ELA groups and in the ELA-L1 and ELA-L2 groups than in the SAL-L1 and SAL-L2 groups.

Increased NFkB activation can result in excessive T-cell activation, which is linked to autoimmune and inflammatory responses^57^. Activated CD4 + cells differentiate into various types of effector T cells (Th1, Th2, Th17, and follicular T cells) that produce cytokines and influence immune responses^58^. Inflammatory Th1 and Th17 cells are closely linked to IFN-γ secretion, which serves as a cellular immune defense and plays a role in inflammatory processes^59^. IL-17, a well-known inflammatory cytokine that attracts monocytes and neutrophils to the site of inflammation, is also released by Th17 cells^60^.

The use of an experimental model of exposure in different locations may have been a limitation for the study, as the animals were transported between the states of SP and ES, and even though it was a short journey, the movement can induce stress and increase the cortisol levels of these animals, and these levels were not measured in the present study.

Nevertheless, the findings of this study emphasize the importance of investigating the effects of particulate matter exposure on lung tissue. This study aimed to better understand these processes by examining inflammation, changes in the extracellular matrix, oxidative stress activation, and the signaling pathways responsible for these lung injury mechanisms.

Cellular expression of iNOS and Gp91ohox were deemed as appropriate marker in this study's evaluation of oxidative stress because elevated levels of this marker indicated oxidative stress in healthy animals and exacerbation in animals with emphysema. Chronic exposure to high concentrations of PM_10_ and PM_2.5_ in the atmosphere, containing substantial amounts of iron and chlorine, is associated with increased pulmonary inflammation due to the absorption of these elements into respiratory tissues. The presence of iron and chlorine in PM particles triggers the formation of reactive oxygen species (ROS) and chlorinated compounds, leading to oxidative stress in the lungs. Oxidative stress, in turn, induces chronic inflammatory responses in pulmonary tissues. Furthermore, prolonged exposure to this combination of atmospheric pollutants can promote pulmonary remodeling, including fibrosis and structural alterations, resulting in a significant deterioration of lung function. This hypothesis suggests that exposure to PM_10_, PM_2.5_, iron and chlorine can initiate a cascade of events that leads to lung damage, including chronic inflammation, oxidative stress, and pulmonary remodeling. This association is relevant for understanding the threat to respiratory health in areas with high air pollution, such as in Vitória, emphasizing the importance of air quality regulation and control, as well as the pursuit of clean energy sources to mitigate these adverse effects.

## Conclusions

In this study, we conclude that iron dust and PM exposures may be responsible for the deterioration of the pulmonary responses in both emphysema and healthy animals. Furthermore, the mechanism involved depends in oxidative stress and inflammation appears to be involve NFkB, iNOS and Gp91phox upregulation, suggesting that oxidative stress mechanisms play a pivotal role in these events.

## Data Availability

The datasets used and/or analyzed during the current study are available from the corresponding author upon reasonable request.
